# Improved MobileNet V3-Based Identification Method for Road Adhesion Coefficient

**DOI:** 10.3390/s24175613

**Published:** 2024-08-29

**Authors:** Binglin Li, Jianqiang Xu, Yufeng Lian, Fengyu Sun, Jincheng Zhou, Jun Luo

**Affiliations:** 1 School of Electrical and Electronic Engineering, Changchun University of Technology, Changchun 130012, China; libinglin@ccut.edu.cn (B.L.); 2202204065@stu.ccut.edu.cn (J.X.); 2202204062@stu.ccut.edu.cn (F.S.); 2202304061@stu.ccut.cn (J.Z.); 2 Vehicle Test Center, Chongqing SERES New Energy Vehicle Design Institute Co., Ltd., Chongqing 401135, China; jun.luo726898@seres.cn

**Keywords:** road adhesion coefficient identification, MobileNet V3, Convolutional Block Attention Module, Bias Loss function, ROS robot platform

## Abstract

To enable the timely adjustment of the control strategy of automobile active safety systems, enhance their capacity to adapt to complex working conditions, and improve driving safety, this paper introduces a new method for predicting road surface state information and recognizing road adhesion coefficients using an enhanced version of the MobileNet V3 model. On one hand, the Squeeze-and-Excitation (SE) is replaced by the Convolutional Block Attention Module (CBAM). It can enhance the extraction of features effectively by considering both spatial and channel dimensions. On the other hand, the cross-entropy loss function is replaced by the Bias Loss function. It can reduce the random prediction problem occurring in the optimization process to improve identification accuracy. Finally, the proposed method is evaluated in an experiment with a four-wheel-drive ROS robot platform. Results indicate that a classification precision of 95.53% is achieved, which is higher than existing road adhesion coefficient identification methods.

## 1. Introduction

**Figure 1:**
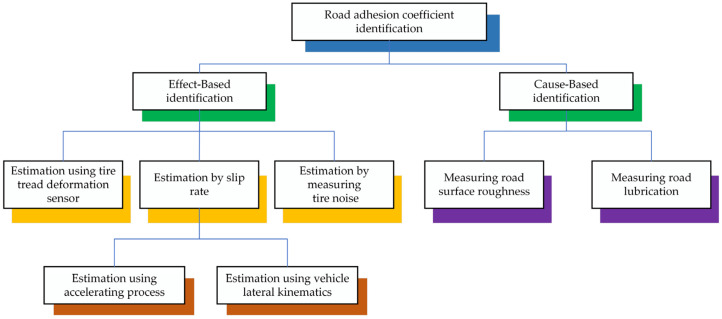
Classification of estimation methods for the road adhesion coefficient.

The rapid development of automotive electronics technology has resulted in the gradual universalization of vehicle active safety control systems in mass-production models, effectively improving vehicle safety. The road surface adhesion coefficient represents a crucial parameter of vehicle active safety control systems, and its precise and expedient estimation can significantly enhance vehicle safety [1]. A reduction in the coefficient of adhesion between the tire and the road surface diminishes the tire’s capacity to adhere to the road surface, which in turn affects the vehicle’s hardware system and its stability control algorithm. Furthermore, this reduces the braking and maneuvering stability of the vehicle, which has a detrimental effect on road traffic safety. Acquiring real-time data concerning the road surface adhesion coefficient is essential for effectively monitoring alterations in road conditions. This information plays a critical role in enhancing vehicle control strategies, ensuring that they are responsive to varying surface conditions. The ability to make precise and trustworthy measurements of adhesion allows for a more agile response to alterations in traction, thereby enhancing road safety and vehicle performance [2,3,4,5]. Despite the challenges associated with directly quantifying this phenomenon using inexpensive and accessible sensor technology, researchers have made noteworthy advancements in this area. In recent years, scientists from around the globe have dedicated substantial resources and expertise to estimating the road adhesion coefficient, leading to the achievement of significant results. These efforts reflect the ongoing commitment to improving our understanding of road conditions and vehicle performance, ultimately contributing to enhanced safety and efficiency in transportation. Methods to determine the road adhesion coefficient can be classified according to their underlying identification principles, which comprise Effect-Based and Cause-Based approaches [6], as illustrated in Figure 1.

The Effect-Based method estimates the road adhesion coefficient by deriving a model from the dynamic response information generated by the vehicle body and tires when driving on different road surfaces. This method does not require the installation of additional sensors. For example, the integration of vehicle dynamics models, refs. [7,8,9,10,11,12] along with tire models and wheel models, plays a crucial role in enhancing the functionality of the Kalman filter [13,14,15,16]. This combination is essential for achieving an accurate determination of the adhesion coefficients present on the road surface, which serves as a reference in the analysis. By utilizing these models within the framework of the Kalman filter, researchers are better equipped to assess and identify the frictional characteristics that impact vehicle performance. For instance, a new adaptive cubature Kalman filter was introduced by Wang et al. [17] for the purpose of estimating the road adhesion coefficient. Hsu et al. [18] developed tables that illustrate the connections among normalized tire longitudinal force, tire slip rate, slip angle, and the tire road adhesion coefficient. Additionally, they suggested a method for estimating the tire road adhesion coefficient for each driving wheel in real-time, utilizing look-up tables. Lin et al. [19] introduced a method of traceless Kalman filtering to enable the real-time assessment of road adhesion coefficients during both straight driving and steering scenarios. Zhang et al. [20] introduced a camber brush tire model that takes into account the characteristics of pressure distribution in the width direction to enhance the precision of adhesion coefficient estimations under camber circumstances. Liang, H. and colleagues [21] could effectively employ 3D laser detection technology to swiftly and accurately evaluate the adhesion of asphalt roads. In the Effect-Based approach, the information gathered for detection was influenced by factors such as the type of tire, tire pressure, the extent of tire wear, and others, resulting in increased noise.

The Cause-Based method employs a priori knowledge, including the type of roadway, to assess the adhesion coefficient of the road [22,23,24,25]. Typically, accelerometer [26] and piezoelectric [27] tread sensors are utilized to track the deflection and vibration of tread components within the contact patch for determining road adhesion coefficients. Zou et al. [28] suggested a technique for assessing the tire-road adhesion coefficient by utilizing an intelligent tire system in conjunction with a three-axis accelerometer. The accuracy of the Cause-Based method in recognition tasks is significantly affected by the level of experience involved in its application. This indicates that practitioners need a substantial amount of training data to develop a reliable understanding of the method’s functioning, as having robust datasets allows for improved learning and adaptation. Consequently, achieving optimal recognition performance often hinges on the depth and breadth of experience amassed through extensive training exercises.

In light of advancements in computational capabilities found in vehicle controllers, alongside the burgeoning field of artificial intelligence, there is increasing enthusiasm surrounding the implementation of deep learning techniques for road recognition. This trend reflects a shift towards leveraging sophisticated algorithms that can enhance the effectiveness of recognition systems. As these technologies continue to evolve, the potential for deep learning methods to improve the accuracy and efficiency of road recognition remains a focal point of research and development in automotive applications. For instance, Roychowdhury and colleagues [29] proposed the machine learning method based on a vision to estimate the road surface attachment coefficient using images captured by a front camera. Liang et al. [30] investigated the performance of road classification methods using Support Vector Machines (SVMs), K-nearest neighbor (KNN), K-Means, ResNet, and MobileNet. The study revealed that deep learning techniques can achieve superior classification accuracy. In conjunction with the requisite specifications for road friction coefficient estimation, Nolte et al. [31] put forth a road condition classification methodology founded upon convolutional neural networks (CNNs). The trained network model was capable of discerning and categorizing six distinct types of roads, including those subjected to snow. Pan et al. [32,33] put forth a CNN-based deep learning model for the recognition and classification of various slippery grades of winter snowy roads. Dewangan et al. [34] developed a deep learning model for road state classification, employing the concept of a CNN, and successfully classified five common road states. Šabanovič et al. [35] developed a deep convolutional neural network that achieved 88.8% accuracy in recognizing six road types, including wet and dry asphalt, wet and dry cobblestone, and wet and dry gravel. Tumen et al. [36] proposed a deep-learning-based method that can be used in vehicle driver assistance systems for automatically recognizing road type and quality. The effectiveness of deep learning algorithms is contingent upon the depth and width of the network. This potential limitation can be addressed by incorporating attention modules, such as the SE module and the CBAM module, which serve to enhance the overall performance. Hnoohom et al. [37] constructed an enhanced deep learning model called SE-ResNet, which uses residual connections and Squeeze-and-Excitation modules to improve the classification performance.

The process of recognizing roads is accomplished through the utilization of a convolutional neural network model. Some of the most prevalent deep learning classification models are AlexNet [38], Visual Geometry Group (VGG) [39], the Residual Neural Network (ResNet) [40], lightweight ShuffleNet V2 [41], and MobileNet V3 [42]. As the depth of the convolutional neural network increases, the accuracy of the recognition task tends to improve. However, this improvement is accompanied by a larger model size and a reduction in operational speed. In light of the vehicle’s necessities for real-time performance and the efficacy of the algorithm, in addition to the actual computational power budget of the in-vehicle embedded system, the selected lightweight image classification network must satisfy the requirements of both high accuracy and high computational speed. Based on this research problem, scholars have proposed lightweight models, such as the MobileNet V3 model, which significantly reduces the model’s size while slightly decreasing its accuracy and increasing its running speed. This methodology has become a prominent area of research, with numerous scholars engaged in its examination.

MobileNet V3 added the SE module, which mainly enhances the attention to the channel but ignores the model’s ability to perceive spatial changes. This paper proposes an improved MobileNet V3 road classification method to address the problem. The approach identifies the road adhesion coefficient by analyzing the relationship between different types of roads and their respective adhesion coefficients. The model employs the well-established deep learning model MobileNet V3 as its foundation, but to enhance precision and minimize misclassification, it has been optimized by substituting the original SE module with the CBAM module. This module incorporates spatial attention while retaining the original channel attention module. This approach enhances the network’s performance by considering both channel and spatial dimensions, thereby allowing it to effectively retrieve more relevant features from these two domains. By optimizing the network in this manner, it gains the ability to identify and utilize more significant information, which leads to improved outcomes in feature extraction. As a result, the network becomes more adept at processing data, which ultimately contributes to achieving better overall results in various applications. Subsequently, the Bias Loss function is employed to supplant the initial cross-entropy loss function within the model, thereby mitigating the potential issues that may arise from random prediction during the optimization process. The model is subsequently trained using the publicly accessible road dataset. The results indicate that it achieves the greatest accuracy in identifying roads. In addition, the suggested model has been deployed on a four-wheel-drive ROS robot.

The main contributions of this study are as follows:

(1) This paper presents an estimation of road adhesion coefficients using a variety of methods. It also provides a detailed account of the well-known work on the estimation of road adhesion coefficients based on image information.
(2) This paper replaces the original SE module with the CBAM. The CBAM not only includes channel attention but also adds spatial attention to optimize the network from both channel and spatial aspects. In this way, the network can be characterized more efficiently from both channel and spatial perspectives.
(3) In this paper, the original cross-entropy loss function in the model is substituted with the Bias Loss function to mitigate the issue of random predictions arising from the optimization process.
(4) The improved model is applied to a four-wheel-drive ROS robot for verification of the model’s validity.

The remainder of the paper is organized as follows: Section 2 outlines the selected models and discusses the proposed extensions. Section 3 provides details on the data sources and results. Finally, Section 4 and Section 5 provide the discussion and conclusions, respectively.

## 2. Materials and Methods

### 2.1. Model Selection

**Figure 2:**
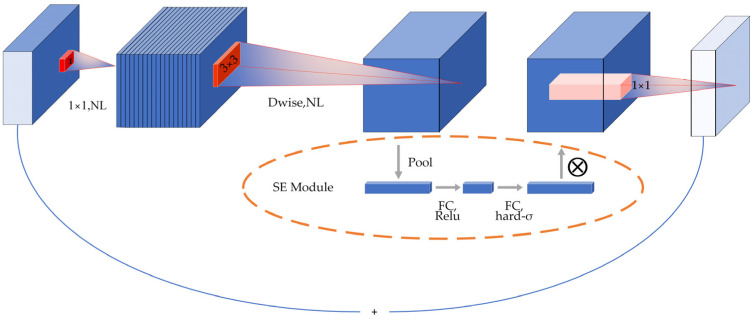
MobileNet V3 block structure.

This research used the lightweight neural network model MobileNet V3. The model, which was introduced by Google in 2019, signifies a substantial advancement by capitalizing on the inherent strengths of MobileNet V1 [43] and MobileNet V2 [44]. It effectively enhances their foundational capabilities, building upon the previous work and innovations established in these earlier versions. This evolution not only reflects the progress in mobile neural network design but also aims to improve efficiency and performance within mobile and edge computing environments. The fundamental component of the MobileNet V3 architecture is depth separable convolution, illustrated in Figure 2. In the figure, NL denotes the nonlinear activation function, ReLU the activation function, and h-swish the activation function, and Dwise denotes the depth-separable convolution. This method divides conventional convolution into two components: convolution performed on each channel separately and pointwise convolution. This results in a significant reduction in model size, which in turn reduces both network parameters and computational complexity. MobileNet V3 includes the SE module, which incorporates a channel focus function to learn the significance of each feature channel. This enables the model to concentrate on relevant feature information and disregard irrelevant information during the image recognition process.

The SE module, introduced in MobileNet V3, is designed to focus on crucial feature information while disregarding irrelevant details in image recognition. By continuously adjusting the weight values, this module enables the precise extraction of essential feature information in diverse scenarios, thereby enhancing the accuracy of the classification process. As illustrated in Figure 2, the primary operation is to perform global average pooling for each channel of the output feature matrix, resulting in the generation of a vector for each channel. Subsequently, the output vectors are obtained through two fully connected layers. In this scenario, the number of vectors in the initial fully connected layer is one-quarter that of the total channels, employing the ReLU activation function. Meanwhile, the output of the second fully connected layer matches the number of channels, utilizing the h-swish activation function. The resulting vector can be interpreted as a weight relation analyzed for each channel-just input, and the resulting weights are weighted to the previous features by multiplying the channels, completing the feature recalibration in the channel dimension.

The precise architecture of the MobileNet V3 model is illustrated in Table 1. In this table, NBN denotes that the batch normalization (BN) component is omitted, RE refers to the ReLU activation function, and HS represents the Hard-swish activation function. The “√” indicates whether the SE Module is incorporated into each layer of the network.

### 2.2. Improvement of the MobileNet V3 Model

The field of feature extraction has experienced significant progress, primarily driven by the continuous advancements in computer vision technology. As the techniques and methodologies within this area have evolved and matured, they have broadened the possibilities and effectiveness of feature extraction processes. Among the pivotal developments that have played a crucial role in this advancement is the introduction of the attention model. This groundbreaking approach has not only improved the accuracy of feature extraction but has also enabled its application across a wide variety of domains, thus enhancing the versatility and utility of feature extraction in numerous contexts. MobileNet V3 added the SE module, which mainly enhances the attention to the channel but ignores the model’s ability to perceive spatial changes. The CBAM [45] combines both channel and spatial attention. Attention feature maps are generated sequentially, traversing both the spatial and channel dimensions. The original input feature map is then multiplied by the resulting two feature maps, producing the final feature map that includes adaptive feature correction. The input feature layer undergoes global average pooling (AvgPool) and global maximum pooling (MaxPool) in the channel attention module. The results of AvgPool and MaxPool are then processed using the Shared MLP. The results that have been obtained are aggregated and analyzed with the application of the Sigmoid activation function to generate the channel attention graph. Subsequently, these weights are utilized on the input feature layer, applied to each channel individually through multiplication. The detailed calculations are presented below.

(1) $$\begin{array}{ll} M_{C}\left(F\right) & =\sigma \left(\mathrm{MLP}\left(\mathrm{AvgPool}\left(F\right)\right)+\mathrm{MLP}\left(\mathrm{MaxPool}\left(F\right)\right)\right)\\ & =\sigma \left(W_{1}\left(W_{0}\left(F_{\mathrm{avg}}^{c}\right)\right)+W_{1}\left(W_{0}\left(F_{\max}^{c}\right)\right)\right) \end{array}$$

where σ denotes the Sigmoid function, $W_{0}\in R^{C\times C/r}$, $W_{1}\in R^{C\times C/r}$, and the weights of the MLP are shared by $W_{0}$ and $W_{1}$.

**Figure 3:**
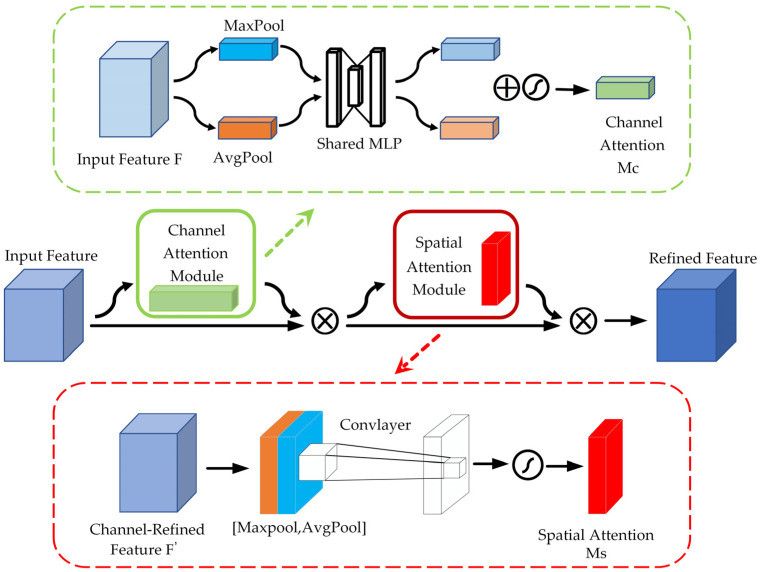
CBAM structure.

The module for spatial attention conducts a global MaxPool of the feature maps for each channel to obtain the maximum response value on each channel. The global maximum response value is mapped to a new feature space through a fully connected layer. The original feature maps are then multiplied by spatial-location-specific weights using an activation function to obtain the adaptively adjusted feature responses. The corresponding spatial locations are then assigned these procedures. Figure 3 illustrates the structure. The specific calculations are shown below.

(2) $$\begin{array}{ll} M_{S}\left(F\right) & =\sigma \left(f^{7\times 7}\left(\left[\mathrm{AvgPool}\left(F\right);\mathrm{MaxPool}\left(F\right)\right]\right)\right)\\ & =\sigma \left(f^{7\times 7}\left(F_{\mathrm{avg}}^{s};F_{\max}^{s}\right)\right) \end{array}$$

where, $F_{\mathrm{avg}}^{s}$ and $F_{\max}^{s}$ refer to the average and maximum pooling of feature *F* under the spatial attention module, respectively. Meanwhile, $f^{7\times 7}$ denotes a 7 × 7 convolution operation.

The Bias Loss function [46] was employed in this study as a substitute for the traditional cross-entropy loss function within the model. This approach aimed to reduce the probability of the model generating arbitrary predictions while being optimized. Such a risk emerges from the limitation of data points, which may not offer an adequate quantity of distinct features to adequately represent the object. Bias Loss functions as a cross-entropy loss that adapts its scaling dynamically. As the variance among the data points diminishes, the scale of the loss progressively reduces. This function enables learning to focus on a large number of unique feature samples, reducing the problem of random prediction during the optimization process. Learning from data points with a large number of unique features can have a positive impact on optimization. The definition of Bias Loss can be found in Equations (3) and (4).

(3) $$L_{\mathrm{bias}}=-\frac{1}{N}\sum_{i=1}^{N}\sum_{j=1}^{k}z\left(v_{i}\right)y_{ij}\log\;f_{j}\left(x_{i};\theta \right)$$

(4) $$z(v_{i})=\exp\left(v_{i}\times \alpha \right)-\beta$$

Let $X\in R^{c\times h\times w}$ be the feature space, where c is a number of input channels, and h, w are the height and width of the input data, and $Y=\left\{1,\ldots ,K\right\}$ be the label space, where K is the number of classes. In a standard scenario, we are given a dataset $D={\left(x_{i},y_{i}\right)}_{i=1}^{N}$ where each $\left(x_{i},y_{i}\right)\in X\times Y$ and a neural network $f\left(X;\theta \right)$. Where θ denotes the model parameters, α and β are tunable contribution parameters and v is the scaled variance of the output of the convolutional layer. The scaling function $z\left(v_{i}\right)$ is nonlinear and creates a bias among data points that exhibit low and high variance.

## 3. Results

### 3.1. Experimental Preparation

**Figure 4:**
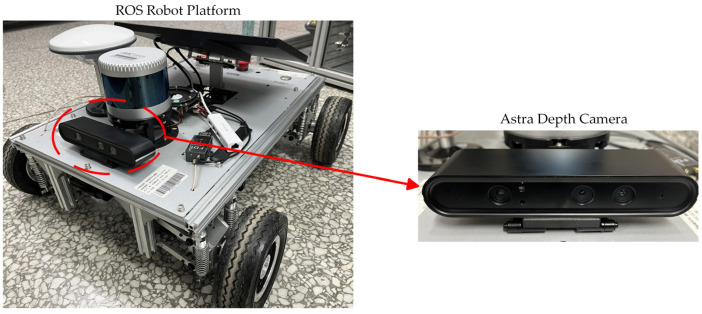
Four-wheel-drive ROS robot platform with an Astra depth camera.

The paper’s experimental environment: a Lenovo P920 tower workstation (Lenovo, Beijing, China), an NVIDIA Geforce RTX3060 12 GB GPU (NVIDIA Corporation, Santa Clara, CA, USA), and configurations such as an Ubuntu 20.04 operating system, Python 3.8 language, CUDA 12.2 parallel computing architecture, and PyTorch 1.12.1 machine learning library. A four-wheel-drive ROS robot platform with an Astra depth camera (ORBBEC, Shenzhen, China) is shown in Figure 4. To assess the model’s performance, detection accuracy, and recall, F1 scores have been chosen as the metrics for evaluating the detection outcomes.

(5) $$\mathrm{Accuracy}=\frac{\mathrm{TP}+\mathrm{TN}}{\mathrm{TP}+\mathrm{FP}+\mathrm{TN}+\mathrm{FN}}$$

(6) $$\mathrm{Precision}=\frac{\mathrm{TP}}{\mathrm{TP}+\mathrm{FP}}$$

(7) $$\mathrm{Recall}=\frac{\mathrm{TP}}{\mathrm{TP}+\mathrm{FN}}$$

(8) $$F1=2\times \frac{\mathrm{Precision}\times \mathrm{Recall}}{\mathrm{Precision}+\mathrm{Recall}}\times 100\%$$

where, the term true positive (TP) refers to the number of samples that have been accurately recognized as positive, whereas false positive (FP) denotes the number of samples mistakenly identified as positive. The false negative (FN) indicates the count of samples wrongly classified as negative, while the true negative (TN) signifies the number of samples that have been correctly labeled as negative.

### 3.2. Dataset

**Figure 5:**
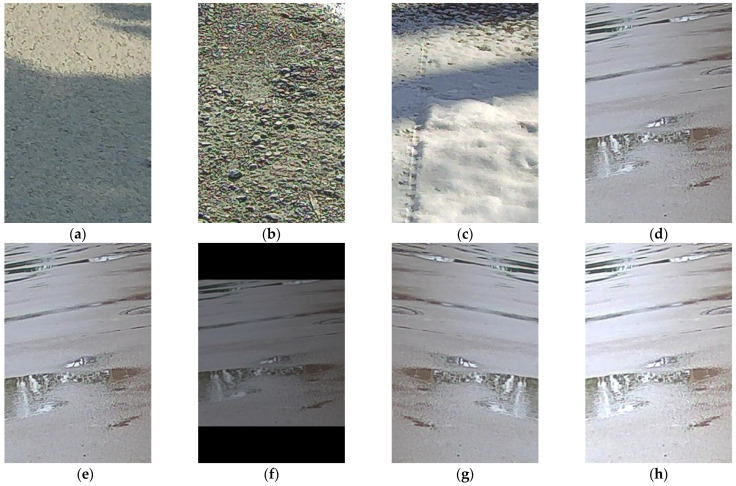
(**a**) For dry-asphalt-smooth, (**b**) for dry-gravel, (**c**) for fresh-snow, (**d**) for water-concrete-smooth, (**e**) for original figure, (**f**) for random cropping, (**g**) for horizontal flip, (**h**) for brightness enhancement.

This study utilized the publicly available dataset RSCD [47] and selected 1000 images of each of the four common types of roads: dry-asphalt-smooth, dry-gravel, fresh-snow, and water-concrete-smooth, totaling 4000 images. The original image resolution was 360 × 224 pixels. This text describes the use of a sample dataset to divide the training and test sets. During model training, data enhancement techniques such as random cropping and horizontal flipping are used to pre-process the training set. These techniques help the model to better learn the different perspectives and variations of the images, which in turn improves the stability of the model training and its generalization ability. Each road sample dataset is shown along with the pre-processed images in Figure 5.

In this paper, refer to “the GA/T643-2006 typical traffic accident pattern vehicle travel speed technical identification” of the automobile longitudinal skid attachment coefficient reference value table and the ice and snow road surface of the automobile longitudinal skid attachment coefficient reference value table [48]. Table 2 displays the median attachment factors from the reference value table for the relevant road type.

### 3.3. Comparison of MobileNet V3 Performance with Other Models

**Figure 6:**
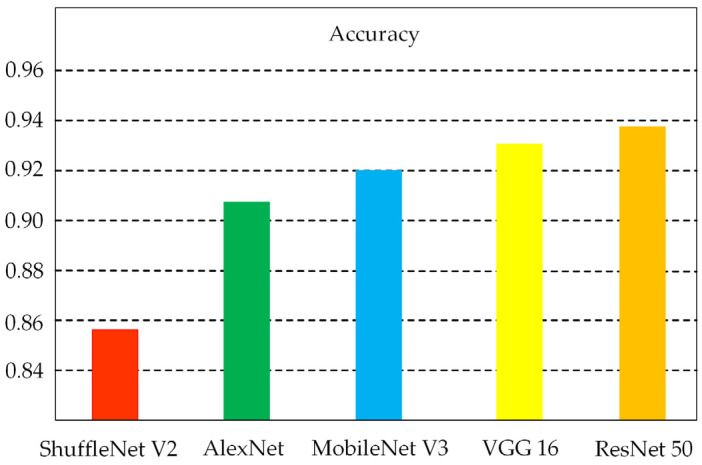
Accuracy of various models.

Table 3 displays the precision, recall, and F1 score for the aforementioned convolutional neural network (CNN) architectures, which are commonly utilized in image processing and classification tasks. The data in the table are based on the road dataset. The results of the experiments suggested that the ResNet 50 architecture employs residual networks that enhance feature extraction and backpropagation capabilities, resulting in superior classification performance during trials. However, this also necessitates increased memory and processing resources. The ShuffleNet V2 employs a technique known as grouped convolution, which serves to reduce the overall number of parameters. However, its convergence process is unstable, which affects the model’s performance. Figure 6 shows the average accuracy of the different models when trained on a dataset containing four different road types. The figure shows that ResNet 50 has the highest accuracy of 93.75%, which is 0.61%, 1.75%, 3.5%, and 6% higher than VGG 16, MobileNet V3, AlexNet, and ShuffleNet V2, respectively. Additionally, the MobileNet V3 model achieves better results with fewer model parameters compared to the other models.

### 3.4. Analysis of Results before and after Model Improvement

Table 4 presents a comparison of the performance metrics of the models before and after the introduction of CBAM and Bias Loss for each type of roadway. The table displays the performance rankings from top to bottom for dry-asphalt-smooth, dry-gravel, fresh-snow, water-concrete-smooth, and average conditions. The MobileNet V3–CBAM model is 0.21 MB larger than the pre-improvement version. Nonetheless, there has been an enhancement of 2.2%, 2.25%, and 2.22% in the model’s mean recognition accuracy, mean recall, and mean F1 score, respectively. The MobileNet V3–CBAM model achieves 100% recognition accuracy for fresh-snow roads with more obvious features. Additionally, it improves the recognition accuracy of water-concrete-smooth roads with less obvious features by 3.3%. The findings indicate that incorporating the CBAM module may enhance the model’s accuracy. When the cross-entropy loss function was substituted with the Bias Loss function in the model, the MobileNet V3–CBAM-Bias model demonstrated average enhancements of 0.93%, 0.98%, and 0.82% in precision, recall, and F1 score, respectively, in comparison to the MobileNet V3–CBAM model. Additionally, compared to the MobileNet V3 model, the MobileNet V3–CBAM model also improved by 3.13%, 3.23%, and 3.04%, respectively. The experimental results indicate that the Bias Loss function can effectively extract more features and also verify the effectiveness of the MobileNet V3–CBAM-Bias model.

**Figure 7:**
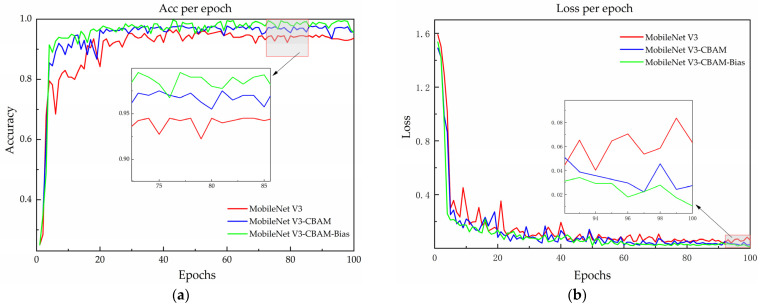
Model accuracy and loss curves before and after improvement (**a**) for a comparison of model accuracy and (**b**) for a comparison of model loss.

The recognition accuracy and loss curves of the models being compared are illustrated in Figure 7 on the validation set. Before the model improvement, the accuracy on the validation set stabilized at around 94% after approximately 40 rounds, and the loss function gradually and smoothly converged to around 0.04 after 30 rounds of iterations. After the implementation of the CBAM module, the validation set accuracy steadily neared 96%, while the loss function stabilized at 0.03 after 20 iterations. The experimental results indicate an improvement in the performance of MobileNet V3–CBAM models.

Substituting the cross-entropy loss function in the model with the Bias Loss function, alongside the integration of the CBAM module, enhances recognition accuracy by 1.75% and reduces the loss value by 1.15% in comparison to MobileNet V3–CBAM. The results demonstrated that introducing a Bias Loss function can mitigate the issue of random prediction during the optimization process. The MobileNet V3–CBAM–Bias model demonstrated a significant improvement in recognition accuracy, surpassing the original model by 3.75%. Furthermore, the loss values were significantly smaller than those of the MobileNet V3 model, decreasing by 2.15%. Although the size of the improved model increased by 0.21 MB, the accuracy of the model was enhanced, and the loss curve decreased and stabilized faster. These results suggest that the MobileNet V3–CBAM–Bias model is more robust. The effectiveness of the improved model has been demonstrated.

**Figure 8:**
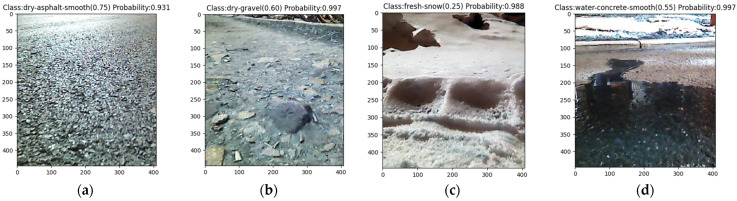
Tests on different road surfaces (**a**) for dry-asphalt-smooth, (**b**) for dry-gravel, (**c**) for fresh-snow, (**d**) for water-concrete-smooth.

The enhanced model was utilized to assess the model’s accuracy on a four-wheel drive ROS robot platform equipped with an Astra depth camera. While controlling the robot to travel at a constant speed, road information is randomly captured and analyzed based on this model, ultimately providing the most probable category information. Figure 8 displays the forecast for various road types. The output provides the road category name, the corresponding road attachment coefficient, and the probability of the category.

## 4. Discussion

The Squeeze-and-Excitation (SE) was replaced by the Convolutional Block Attention Module (CBAM). The model includes a spatial attention component alongside the existing channel attention module, facilitating the concurrent extraction of features from both spatial and channel viewpoints. Subsequently, the Bias Loss function substitutes the cross-entropy loss function in the base model, allowing for the extraction of a larger number of features, which in turn improves the model’s recognition accuracy. This approach was evaluated on the publicly accessible RSCD dataset, and the findings indicated that the enhanced model exhibited a 0.21 MB increase in size. The accuracy, recall, and F1 value were enhanced by 3.13% in comparison to the base model while maintaining a lightweight structure. The respective enhancements were 3.23% and 3.04%. The findings indicated that the enhanced model demonstrated significant effectiveness and superiority in identifying road images, achieving greater speed and accuracy. A new approach is provided for the recognition of road adhesion coefficients.

## 5. Conclusions

This paper employs deep learning techniques to address the challenge of recognizing diverse road surfaces and their corresponding attachment coefficients. The objective was to anticipate changes in road conditions and provide the requisite information for effective vehicle control. The accurate estimation of the road surface attachment coefficient was conducive to the enhancement of automobiles’ self-adaptive capabilities in response to environmental stimuli, thereby optimizing the utilization of Intelligent Transport Systems (ITSs) and facilitating the real-time adjustment of driving speed, which is of paramount importance for the advancement of driving-environment-sensing systems and vehicle stability control algorithms. The ability to predict the condition of the road surface in advance and to accurately estimate the attachment coefficient is conducive to the automobile active safety system adjusting the control strategy in a timely manner, enhancing adaptability to complex working conditions, and further improving the safety of driving. In order to tackle this issue, the present paper introduces an improved deep learning model that utilizes the MobileNet V3 framework. In addition to developing a model for road surface recognition, this study also implemented it on a four-wheel-drive ROS robot platform for validation. The experimental results showed that the improved model is very effective and superior in recognizing road images with faster speed and higher accuracy.

In the future, the variety of road types will be expanded to accommodate a greater range of roads. To enhance the precision of the model while minimizing the number of parameters, the MobileNet V3 architecture will be optimized and evaluated in comparison with more contemporary models to guarantee the generalizability of this study. Furthermore, the robustness of the model will be enhanced in practical deployment to account for the influence of external factors. The subsequent phase of the research will entail an investigation into the deployment of more sophisticated and prevalent model architectures, with the objective of reducing the impact of the surrounding environment on the accuracy of pavement recognition.

## Figures and Tables

**Table 1:** Network structure of the MobileNet V3 model.

Operator	Input Channel	Size	SE Module	NL	Step
ConvBNA, 3 × 3	3	224 × 224	-	HS	2
InvertedResidual, 3 × 3	16	112 × 112	-	RE	1
InvertedResidual, 3 × 3	16	112 × 112	-	RE	2
InvertedResidual, 3 × 3	24	56 × 56	-	RE	1
InvertedResidual, 5 × 5	24	56 × 56	√	RE	2
InvertedResidual, 5 × 5	40	28 × 28	√	RE	1
InvertedResidual, 5 × 5	40	28 × 28	√	RE	1
InvertedResidual, 3 × 3	40	28 × 28	-	HS	2
InvertedResidual, 3 × 3	80	14 × 14	-	HS	1
InvertedResidual, 3 × 3	80	14 × 14	-	HS	1
InvertedResidual, 3 × 3	80	14 × 14	-	HS	1
InvertedResidual, 3 × 3	80	14 × 14	√	HS	1
InvertedResidual, 3 × 3	112	14 × 14	√	HS	1
InvertedResidual, 5 × 5	112	14 × 14	√	HS	2
InvertedResidual, 5 × 5	160	7 × 7	√	HS	1
InvertedResidual, 5 × 5	160	7 × 7	√	HS	1
Conv2d, 1 × 1	160	7 × 7	-	HS	1
Avg Pooling, 7 × 7	960	7 × 7	-	-	1
Conv2d, 1 × 1, NBN	960	1 × 1	-	HS	1
Conv2d, 1 × 1, NBN	1280	1 × 1	-	-	1

**Table 2:** Corresponding values of road type and road adhesion coefficient.

	Dry- Asphalt-Smooth	Dry- Gravel	Fresh- Snow	Water- Concrete-Smooth
Road adhesion coefficient	0.75	0.60	0.55	0.25

**Table 3:** Comparison of MobileNet V3 performance with other models.

	Precision (%)	Recall (%)	F1 Score (%)	Model Size (M)
ResNet 50	94.13	93.75	93.94	25.24
VGG 16	93.65	93.14	93.40	138.18
AlexNet	90.83	90.25	90.54	16.68
MobileNet V3	92.40	92.00	92.20	5.48
ShuffleNet V2	88.50	87.50	87.99	7.16

**Table 4:** Comparison performance before and after model improvement.

	Model Size (M)	Road Type	Precision (%)	Recall (%)	F1 Score (%)
MobileNet V3	5.48	dry-asphalt-smooth	94.80	91.00	92.86
		dry-gravel	92.80	90.00	91.38
		fresh-snow	97.90	92.00	94.86
		water-concrete-smooth	84.10	95.00	89.22
		average	92.40	92.00	92.20
MobileNet V3–CBAM	5.69	dry-asphalt-smooth	96.90	94.00	95.43
		dry-gravel	94.10	95.00	94.55
		fresh-snow	100.0	91.00	95.29
		water-concrete-smooth	87.40	97.00	91.95
		average	94.60	94.25	94.42
MobileNet V3–CBAM-Bias	5.69	dry-asphalt-smooth	98.90	94.00	96.39
		dry-gravel	94.90	97.90	96.38
		fresh-snow	100.0	91.00	95.29
		water-concrete-smooth	88.30	98.00	92.89
		average	95.53	95.23	95.24

## Data Availability

The data that support the findings of this study are openly available on RSCD.
